# Discovery of Novel Epoxyketone Peptides as Lipase Inhibitors

**DOI:** 10.3390/molecules27072261

**Published:** 2022-03-31

**Authors:** Jehad Almaliti, Muhammed Alzweiri, Momen Alhindy, Tamam Al-Helo, Ibrahim Daoud, Raghad Deknash, C. Benjamin Naman, Bashaer Abu-Irmaileh, Yasser Bustanji, Islam Hamad

**Affiliations:** 1 Department Pharmaceutical Sciences, College of Pharmacy, The University of Jordan, Amman 11942, Jordan; m.alzweiri@ju.edu.jo (M.A.); m.alhindi@ju.edu.jo (M.A.); tmam.helo@gmail.com (T.A.-H.); ibrahim.mahsery@gmail.com (I.D.); rgd0166052@ju.edu.jo (R.D.); bustanji@ju.edu.jo (Y.B.); 2 Skaggs School of Pharmacy and Pharmaceutical Sciences, University of California San Diego, La Jolla, San Diego, CA 92093, USA; 3 Li Dak Sum Yip Yio Chin Kenneth Li Marine Biopharmaceutical Research Center, Department of Marine Pharmacy, College of Food and Pharmaceutical Sciences, Ningbo University, Ningbo 315800, China; bnaman@nbu.edu.cn; 4 Hamdi Mango Center for Scientific Research, The University of Jordan, Amman 11942, Jordan; bashaeraburmaileh@yahoo.com; 5 Department of Basic Medical Sciences, College of Medicine, University of Sharjah, Sharjah P.O. Box 27272, United Arab Emirates; 6 Department of Pharmacy, Faculty of Health Sciences, American University of Madaba, Madaba 11821, Jordan; i.hamad@aum.edu.jo

**Keywords:** lipase inhibitors, novel warhead, epoxyketone, dyslipidemia, obesity, peptide inhibitors

## Abstract

Obesity is the most common nutritional disorder in the developed world and is associated with important comorbidities. Pancreatic lipase (PL) inhibitors play a key role in the metabolism of human fat. A series of novel epoxyketones peptide derivatives were investigated for their pancreatic lipase inhibitory activity. The epoxyketone moiety is a well-known reactive electrophile group that has been used as part of proteasome inhibitors in cancer therapy, and it is widely believed that these are very selective for targeting the proteasome active site. Here we investigated various peptide derivatives with an epoxide warhead for their anti-lipase activity. The assessment of these novel epoxyketones was performed by an in-house method that we developed for rapid screening and identification of lipase inhibitors using GC-FID. Herein, we present a novel anti-lipase pharmacophore based on epoxyketone peptide derivatives that showed potent anti-lipase activity. Many of these derivatives had comparable or more potent activity than the clinically used lipase inhibitors such as orlistat. In addition, the lipase appears to be inhibited by a wide range of epoxyketone analogues regardless of the configuration of the epoxide in the epoxyketone moiety. The presented data in this study shows the first example of the use of epoxyketone peptides as novel lipase inhibitors.

## 1. Introduction

Obesity is a serious and burdensome health disease that has persisted for many years and affected the lives of significant number of the population worldwide [1,2]. Obesity is associated with serious comorbidities, including diabetes (type 2), hypertension, dyslipidemia, sleep apnea, arthritis, hyperuricemia, gall bladder disease, certain types of cancer, and it is closely related to the emergence of many chronic diseases [1]. Obesity can also lead to abnormal metabolism, which causes series of physiological, psychological, and social problems [3]. In fact, recently it was found that obesity increases the risk of severe illness from COVID-19 and it may even triple the risk of hospitalization due to COVID-19 infection [4,5]. This is believed to be due to links between obesity and immune system impairments. Consequently, the prevention and treatment of obesity itself is an important factor for reducing the prevalence and mortality of various chronic diseases. The current treatments of obesity suffer from low efficacy and long-term drug treatment regimen combined with long-term diet plus exercise lifestyle modifications to achieve weight loss [6]. The only drug presently marketed as a long-term obesity treatment is orlistat (Xenical^®^; Roche), which works as an inhibitor of nutrient digestion and absorption through inhibition of gastric and pancreatic lipase [7]. By using anti-obesity agents that act as potent lipase inhibitor medications together with a reduced-calorie diet and increased physical activity, significant weight loss can be achieved in most of these patients. Other less-favored treatment approaches work either by altering brain chemistry or enhancing of energy expenditure to act peripherally and increase lipid oxidation or thermogenesis [8,9]. Hence, the lipase represents an attractive target for drug development for the treatment of obesity.

**Figure 1:**
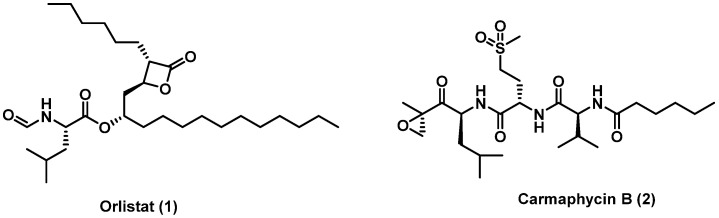
Chemical structure of orlistat (**1**) and carmaphycin B (**2**).

Lipases are the key digestive enzymes of the GI tracts that are responsible for the hydrolysis of dietary fats into small lipid molecules, triglycerides, glycerol, and free fatty acids [10]. Some lipases degrade a broad range of ester compounds and hydrolyze the ester bonds in acylglycerols; hence, these enzymes can lack acute substrate specificity and have the ability to use a wide range of structurally diverse nonnatural compounds as substrates [11]. Pancreatic lipase (PL) is one of the key role lipases in food fat digestion and absorption [12]. PL is a serine hydrolase that is required to efficiently digest triglyceride at the sn1 position and release 2-monoacylgycerols and free fatty acids. Therefore, PL plays a vital role in food fat digestion and absorption. One of the major strategies to control obesity is the inhibition of PL. Orlistat (**1**; Figure 1), a site-directed β-lactone inhibitor of human digestive lipases, is the only FDA approved drug for the treatment of obesity that works through this mechanism. However, the use of orlistat is hampered by low selectivity and off-target activity on serine hydrolases other than lipases, and many patients reported serious side effects including hepatotoxicity and acute pancreatitis [13,14]. In addition, the therapeutic dose of orlistat only inhibits dietary fat absorption by approximately 30% [15]. Hence, there is a need for more selective and effective medications that can better inhibit metabolism of dietary fats with less risk of systemic toxicity. 

Peptide epoxyketones are known as potent covalent proteasome inhibitors [16]. Epoxyketones are known to be selective inhibitors for proteasome, which is controlled by the epoxyketone electrophilic warhead [17]. The cyanobacterial natural product carmaphycin B (**2**; Figure 1) is an example of these compounds, and it shares some structural features with the FDA approved drug carfilzomib [18]. The epoxyketone reacts specifically with the *N*-terminal threonine residue of the active proteasome subunit [19]. However, this warhead of the epoxyketones was not explored previously for their lipase inhibitory activity. Herein described is our newly developed library of novel epoxyketone peptides that showed potent anti-lipase activity. This is the first example of any epoxyketone covalent inhibitor of PL, which represents a new pharmacophore that can be explored for potent anti-lipase inhibitory activity and further drug development. Many of the synthesized derivatives showed anti-lipase activity comparable to or more potent than the clinically used lipase inhibitor orlistat (**1**; Figure 1).

## 2. Results and Discussion

**Figure 2:**
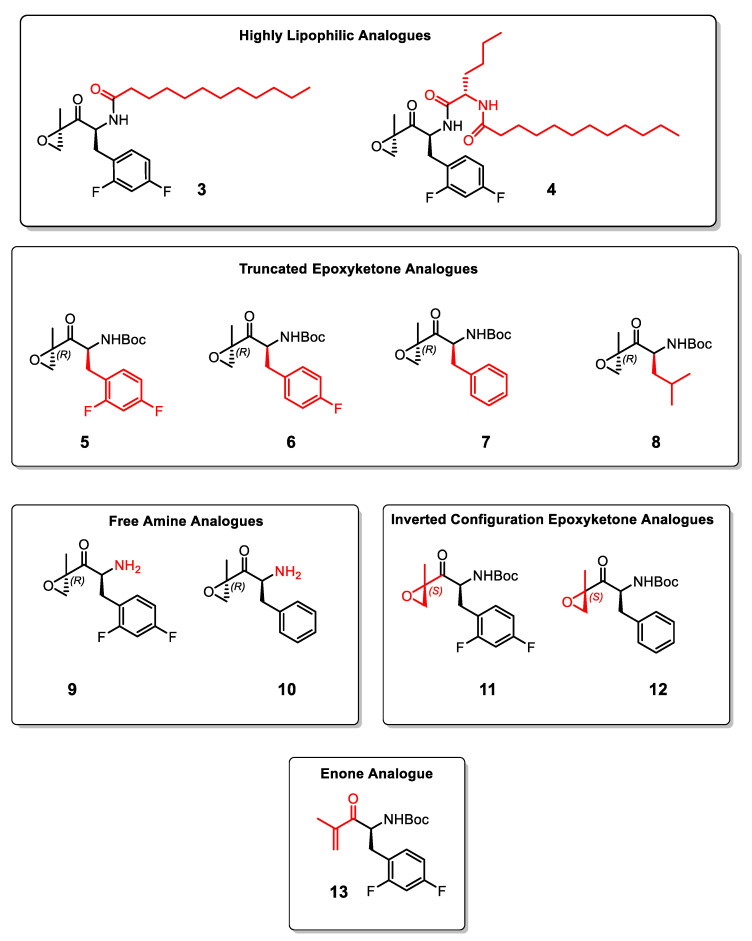
The designed and synthesized library of epoxyketone analogues **3**–**12** and enone **13**.

We hypothesized that the serine residues in the active site of PL can bind to the epoxide of the epoxyketone due to its strong electrophilic characteristic, especially since lipases are known to catalyze the degradation of a broad range of electrophiles including esters. To initially test this hypothesis, we used the natural product carmaphycin B (**2**; Figure 1). We found that this epoxyketone peptide inhibited porcine PL potently with IC_50_ = 25 nM. The relevance of this was further demonstrated using orlistat (**1**) as a positive control, which had IC_50_ = 100.8 nM, similar to the reported value in the literature [20]. Hence, carmaphycin B with its epoxyketone warhead showed more potent PL inhibitory activity than the FDA approved drug, orlistat. However, carmaphycin B (**2**) is also known to efficiently inhibit the human constitutive proteasome to such extent that its use as an anti-obesity agent must be counter-indicated due to predictable cytotoxicity and serious side effects [18,21]. This encouraged us to design and synthesize a suite of structural analogues that mostly retain the epoxyketone warhead (Figure 2). The goal was, most importantly, to have analogues that inhibit selectively the PL but not the human constitutive proteasome to reduce or prevent serious clinical side effects in future development. These analogues include more lipophilicity than carmaphycin B (i.e., analogues **3** and **4**), to simulate the high lipophilicity of orlistat and triglycerides, and a truncated version of the carmaphycin B to reduce the peptidic nature, proteasome substrate acceptance, and to balance the lipophilic/hydrophilic property of these molecules (i.e., analogues **5**–**10**). Orlistat is known to act mainly via its local effect in the gut, and systemic exposure to the medication is minimal [15]. Thus, protected and free amine forms of these analogues were explored to modulate the solubility of these compounds and reduce their capacity for systematic absorption (analogues **9**–**10**).The epoxyketone moiety was also replaced with an enone moiety to evaluate the inhibitory activity of a different electrophilic group (analogue **13**). Lastly, after synthesizing both configurations of the epoxyketone moiety in these compounds (analogues **11**–**12**), the impact of stereochemistry of the epoxyketone on pharmacology was investigated in relation to their epimers (**5** and **7**).

Analogue **4** represents a truncated and very lipophilic version of **2**, and it is composed of lauric acid, norleucine, and 2,4-difluoro-l-phenylalanine-epoxyketone. This compound very potently inhibits porcine PL with IC_50_ = 9.3 nM (Table 1). The highly lipophilic character of this analogue (CLogP = 8.7) was designed to simulate the lipophilic nature of the natural triglyceride ligands as well as orlistat (CLogP = 8.6). However, systemic absorption would also likely be enhanced for this lipophilic analogue, which might cause undesired side effects if other lipases are inhibited as well. A systemic inhibition of lipases might achieve different and less favorable clinical anti-obesity outcomes [10]. Therefore, compound **3** was designed with less lipophilic characteristic by eliminating the lipophilic norleucine moiety in compound **4**. Analogue **3** was made with lauric acid, and 2,4-difluoro-l-phenylalanine-epoxyketone, is a simplified analogue of **4** with CLogP = 6.3, and it has also shown comparable and still potent IC_50_ against PL (29.3 nM).

We have also evaluated the activity of the warhead amino acid-epoxyketone alone without the fatty side chain, as in analogues **5**–**12**, to assess the effect of removing other terminal moieties (norleucine or lauric acid) on the lipase inhibitory activity. To our surprise, the Boc-protected analogues **5**–**8**, and the free amine analogues **9**–**10** were all active against the PL (Table 1). The Boc-protected analogues **5**–**8** have shown slightly more potency against PL lipases compared to the free amine analogues **9**–**10**, possibly due to the preferred lipophilic nature of these analogues with the PL enzyme. In addition, analogue **5**, which contains the 2,4-difluoro-l-phenylalanine as side chain, has shown 1.5-to−2-fold increased potency toward inhibiting the pancreatic lipase compared to analogues **6** and **7** that contain 4-fluoro-l-phenylalanine and phenylalanine in the side chains, respectively. This slight improvement in potency is likely due to the presence of the fluorine in analogue **5**, which improves the lipophilic characters of this analogue and adds an additional H-bond element in the structure. The non-aromatic leucine analogue **8** had lower potency than the aromatic side chain analogues **5**–**7**.

**Figure 3:**
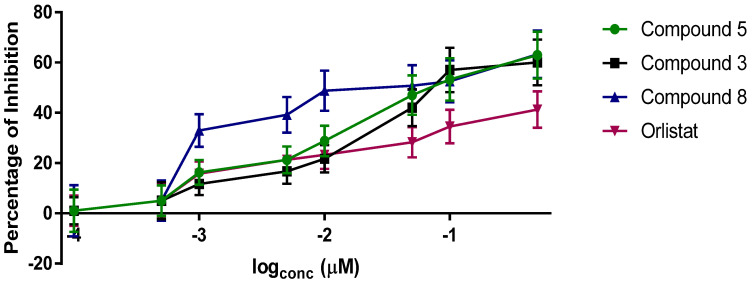
Inhibition curves for orlistat (**1**), compounds **3**, **5**, and **8** with the PL.

By inverting the configuration of the epoxide present in analogues **5** and **7** from *R* to *S*, we have produced analogues **11** and **12,** which surprisingly showed similar potency against PL with IC_50_ values of 29.0 and 49.2 nM, respectively (Table 1). This remarkable finding suggests that the lipase enzymes are not selective based on the stereochemistry of the warhead in this pharmacophore and that they can target a wide array of electrophiles. In contrast, the human constitutive proteasome is selectively inhibited by the *R*-isomer of the epoxyketone and does not bind to the inverted *S*-isomer of the epoxyketone. Hence, the *R*-isomer analogues **11**–**12** are anticipated to have improved selectivity toward the PL over the human constitutive proteasome and thus lower undesired cytotoxicity and off-target side effects. We have additionally synthesized analogue **13**, which contains an enone warhead instead of the epoxyketone warhead in compounds **5** and **11.** This compound did not inhibit PL at the highest concentration tested (10 µM), which demonstrates the importance of using epoxyketone as a warhead to inhibit the PL active site irreversibly. The weak electrophilic nature of the enone in **13** is possibly the reason for inactivity against the lipases compared to the epoxyketones. Figure 3 depicts IC_50_ curves of orlistat and some selected compounds generated in this study (**3**, **5**, and **8**), which shows that these three analogues are more potent PL inhibitors than the currently used drug, orlistat.

**Figure 4:**
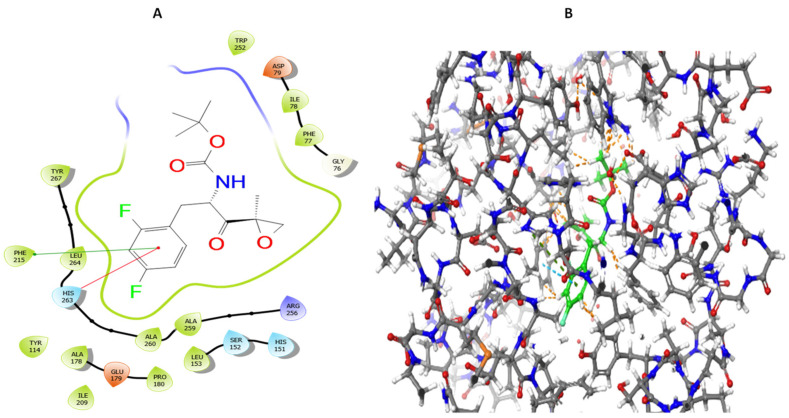
Representations of the 2D (**A**) and 3D (**B**) interactions of compound **5** within the active site of human pancreatic lipase before the formation of irreversible binding.

The mechanism of orlistat inhibition against lipase enzyme is irreversible due to a covalent bond that results from the nucleophilic attack of the hydroxyl group side chain of serine 152 on the orlistat lactone ring [22,23]. However, the physical access via “protein binding” of the candidate inhibitor to the active site of the enzyme is essential to introduce the inhibitor in the vicinity of the serine residue in the active site. Therefore, docking screening of the compounds was carried out for orlistat and our synthesized compounds. The synthesized compounds depicted docking scores at least twice that of orlistat. Notably, it has been reported previously that low values of docking scores for orlistat do not correlate well with its potency against lipase enzymes [22,23]. This might be explained by the excessive conformational flexibility of orlistat alkyl chains which reduces its affinity to bind within the lipase active site or overpopulates the conformer library and masks the most active conformer(s). Additionally, docking may predict the competitive binding satisfactorily, but it has a marginal benefit in evaluating irreversible non-competitive binding. However, the physical binding of orlistat with lipase is a step necessary to adjoin the drug with the nucleophilic residue in the enzymatic active site. As depicted in Figure 4**,** compound **5** embodies the physical interaction with the PL active site before the covalent bond formation between serine 152 and the strained epoxide ring in the molecule. There is Π-Π stacking observed between the substituted phenyl ring in **5** with the side chains of phenylalanine 215 and histidine 263. Interestingly, the phenol of tyrosine 267 can act as a hydrogen bond donor to one of the fluorine atoms in **5**, and, consequently, this improves the ligand binding with the enzyme. However, it is important to emphasize that the activity not only relies on the ligand binding with the lipase, but also on the reactivity of the epoxide ring and its accessibility to the serine residue inside the active site cavity. Noticeably, three member rings such as epoxides usually possess sufficient ring strain from angle, torsion, and steric effects to render them quite reactive as alkylating agents.

Since the epoxyketone moiety represents an important part of many anticancer agents that target the human constitutive proteasome, the activity of these analogues were also evaluated against two different human cell lines (Table 2), the CCD-1064Sk fibroblast cell line and the human hepatoma cell line HepG2 [24,25]. This is crucial to show whether these compounds could be well-tolerated or if they would likely cause off-target side effects due to an undesired inhibition of the human constitutive proteasome or other broadly cytotoxic mechanisms. The fibroblasts cell line CCD-1064Sk was chosen to evaluate the cytotoxic effect of these compounds on normal human cells, while the HepG2 cells are widely used cancer cell lines to evaluate the proteasome inhibitors due to their known sensitivity to this class of compounds [25]. Many of the designed analogues had low-to-no inhibitory activity against these cells, indicating that these compounds are not targeting the human constitutive proteasome as the lead compound, carmaphycin B. In fact, our previous work in this field demonstrated that shortened versions of carmaphycin B designed by removing any of the amino acids or the *N*-cap group, or inverting configuration in the epoxyketone resulted in drastic reduction in the proteasome inhibitory activity and significant reduction in the cytotoxicity as a result. Consequently, these compounds that have shown selective inhibitory for the lipase enzyme are understood to hold great potential to be developed as anti-obesity drug leads with a novel pharmacophore and predicted negligible toxicity. 

## 3. Materials and Methods

### 3.1. Materials and Instrumentations

All chemicals and solvents were purchased from commercial suppliers (Amman, Jordan) and used without further purification. Anhydrous solvents were distilled from sodium and benzophenone before use. NMR (The University of Jordan, Amman, Jordan) spectra were recorded on a Varian 500 MHz spectrometer (500 and 125 MHz for the ^1^H and ^13^C nuclei, respectively) using CDCl_3_ as solvents and spectra were referenced to residual solvent as internal standard (for CDCl_3_ δH 7.26 and δC 77.1). LC-HRMS data for analysis of compounds **3**−**13** were obtained on an Agilent 6239 HR-ESI-TOFMS (UCSD, San Diego, CA, USA) equipped with a Phenomenex Luna 5 µm C18 100 Å column (4.6 × 250 mm). LCMS data for purity analysis of the synthesized compounds 3−13 were obtained with a Thermo Finnigan Surveyor Autosampler-Plus/LC-PumpPlus/PDA-Plus system and a Thermo Finnigan LCQ Advantage Max mass spectrometer (monitoring *m/z* 150−2000 in positive ion mode) using a linear gradient of 30–100% H_2_O/acetonitrile over 30 min; flow rate of 0.9 mL/min (UCSD, San Diego, CA, USA). Semipreparative HPLC (UCSD, San Diego, CA, USA) purification was carried out using a Waters 515 with a Waters 996 photodiode array detector using Empower Pro software. Structural integrity and purity of the test compounds were determined by the composite of 1H and 13C NMR, HRMS and HPLC, and all compounds were found to be >93% pure. Chemical shifts (δ) are given in parts per million (ppm) and coupling constants (*J*) are reported in Hertz (Hz). The compounds are named in accordance with IUPAC rules as applied by ChemBioDraw Ultra (version 20.1.1) (software provided by the University of Jordan, Amman, Jordan). GCMS analysis: Thermo Scientific Focus gas chromatography coupled to a flame ionization indicator was used to analyze the samples. A total of 1.0 µL sample was injected into the Thermo Scientific Focus GC-FID equipped with a split/spitless injector (split ratio, 2.5). The column was HP-5 (DP-5) capillary column stationary phase. Helium was the carrier gas at a flow rate of 1.0 mL/min. Linear temperature program was applied from 100 to 250 °C at 5 °C/min. The specific run time of analysis for each sample was 15 min. The temperatures of the injector base and the detector were maintained at 250 °C, 250 °C respectively. Lipase from porcine pancreas, Type II, 100–650 units/mg protein (product# L3126) was purchased from Sigma Aldrich (Amman, Jordan). 

### 3.2. Synthesis

**Analogues 3**–**13 were synthesized according to the following procedures:** [25,26]

**Synthesis of the phenylalanine enone compound 13.**

To a solution of Boc-Phe-OH (1 equiv.), NH(Me)OMe·HCl (1.5 equiv.), and HBTU (1.5 equiv.) in DCM (0.28 M), was added DiPEA (3.0 equiv.). The solution was stirred at room temperature for overnight, and after TLC showed completion of reaction, the solvent was removed under reduced pressure and the residue was resuspended in ethyl acetate. The organic layer was washed with 1 M HCl (3X), saturated NaHCO_3_ (2X), brine, and then dried using anhydrous magnesium sulfate. It was evaporated under vacuum and the crude product was purified by column chromatography (25–75% ethyl acetate/hexanes) to give the Weinreb amides in quantitative yield as colorless oils. 

To a solution of the Weinreb amides (1 equiv.) in THF (0.34 M) at 0 °C was added isopropenyl magnesium bromide solution (2.0 equiv., 0.5 M solution in THF) drop wise and the reaction was stirred at 0 °C overnight. After TLC showed completion of the reaction, ice-cold saturated aqueous ammonium chloride solution was added followed by ethyl acetate. The organic layer was washed with brine, dried over anhydrous magnesium sulfate and evaporated. The crude product was purified on column chromatography (25–75% ethyl acetate-hexanes) and the product **13** was obtained as a colorless oil (yields 68%).

***tert-butyl (S)-(1-(2,4-difluorophenyl)-4-methyl-3-oxopent-4-en-2-yl)carbamate*** (**13**). The product was synthesized according to the published procedures [25]. ^1^H NMR (500 MHz, CDCl_3_) δ 7.11–6.99 (m, 1H), 6.89–6.67 (m, 2H), 6.10 (s, 1H), 5.92 (s, 1H), 5.37–5.25 (m, 1H), 3.14 (dd, *J* = 14.1, 4.3 Hz, 1H), 2.81 (dd, *J* = 13.9, 5.8 Hz, 1H), 1.89 (s, 3H), 1.37 (s, 9H).; ^13^C NMR (125 MHz, CDCl_3_) δ 199.68, 163.41, 161.44, 155.19, 142.48, 132.69, 132.64, 119.40, 111.34, 103.94, 79.95, 33.35, 28.48, 17.87. ESI-HRMS [M + H]^+^ *m/z* 324.1619 (calculated for C_17_H_22_F_2_NO_3_ is 324.1611).

**General Synthesis procedure for the epoxyketones 5**–**12.**

A solution of previously prepared enone (1 equiv.) in DMF (0.3 M) was cooled to −20 °C, and bleach (10% concentration, 2 equiv.) was added dropwise to avoid temperature change and then the reaction mixture was warmed to 0 °C and stirred for 2 h. Water was added to the reaction mixture, and the mixture was extracted with ethyl acetate. The organic phases were combined, washed with brine, dried over anhydrous magnesium sulfate, and concentrated. The resulted residue was purified by flash column chromatography on silica gel to afford the desired epoxyketone compounds (compounds **5**–**8** and **11**–**12**) as viscous oil (4:1 ratio of epoxide diastereomers). The Boc protecting group was removed by dissolving the desired epoxyketone product in DCM:TFA (2:1) for 20 min, and the solvent evaporated to give the corresponding TFA amine salt product **9**–**10**.

***tert-butyl ((S)-3-(2,4-difluorophenyl)-1-((R)-2-methyloxiran-2-yl)-1-oxopropan-2-yl)carbamate*** (**5**). The product was synthesized according to the general procedure. ^1^H NMR (500 MHz, CDCl_3_) δ 7.13 (q, *J* = 7.9 Hz, 1H), 6.82–6.80 (m, 2H), 5.03 (d, *J* = 8.7 Hz, 1H), 4.56 (q, *J* = 7.1 Hz, 1H), 3.26 (d, *J* = 4.9 Hz, 1H), 3.03–2.94 (m, 2H), 2.93 (d, *J* = 5.2 Hz, 1H), 1.53 (s, 3H), 1.38 (s, 9H); ^13^C NMR (125 MHz, CDCl_3_) δ 207.9, 163.4, 162.5, 161.4, 160.5, 155.3, 132.5, 118.9, 111.6, 103.8, 80.2, 59.3, 52.9, 52.6, 30.5, 28.4, 16.6. ESI-HRMS [M + H]^+^ *m*/*z* 342.1518 (calculated for C_17_H_22_F_2_NO_4_ is 342.1517).

***tert-butyl ((S)-3-(4-fluorophenyl)-1-((R)-2-methyloxiran-2-yl)-1-oxopropan-2-yl)carbamate*** (**6**). The product was synthesized according to the general procedure. ^1^H NMR (500 MHz, CDCl_3_) δ 7.40 (d, *J* = 8.3 Hz, 2H), 7.02 (d, *J* = 8.1 Hz, 2H), 4.92 (d, *J* = 8.5 Hz, 1H), 4.53 (td, *J* = 8.2, 4.8 Hz, 1H), 3.25 (d, *J* = 4.9 Hz, 1H), 3.05 (dd, *J* = 14.0, 4.8 Hz, 1H), 2.90 (d, *J* = 4.9 Hz, 1H), 2.65 (dd, *J* = 14.0, 7.9 Hz, 1H), 1.49 (s, 3H), 1.35 (s, 9H); ^13^C NMR (125 MHz, CDCl_3_) δ 208.1, 155.2, 135.0, 131.6, 131.2, 121.0, 80.1, 59.1, 53.5, 52.5, 37.0, 28.3, 16.6. ESI-HRMS [M + H]^+^ *m*/*z* 324.1617 (calculated for C_17_H_23_FNO_4_ is 324.1611).

***tert-butyl ((S)-1-((R)-2-methyloxiran-2-yl)-1-oxo-3-phenylpropan-2-yl)carbamate*** (**7**). The product was synthesized according to the general procedure. ^1^H NMR (500 MHz, CDCl_3_) δ 7.31 (dd, *J* = 8.3, 6.6 Hz, 2H), 7.28–7.24 (m, 1H), 7.17 (d, *J* = 7.5 Hz, 2H), 4.95 (d, J = 8.3 Hz, 1H), 4.59 (td, *J* = 8.0, 5.1 Hz, 1H), 3.29 (d, *J* = 4.9 Hz, 1H), 3.11 (dd, *J* = 13.9, 5.0 Hz, 2H), 2.91 (d, *J* = 4.9 Hz, 1H), 2.74 (dd, *J* = 14.0, 7.8 Hz, 1H), 1.51 (s, 3H), 1.37 (s, 9H); ^13^C NMR (125 MHz, CDCl_3_) δ 208.6, 155.4, 136.1, 129.6, 128.7, 127.2, 80.0, 59.4, 53.8, 52.6, 37.7, 28.5, 16.8. ESI-HRMS [M + H]^+^ *m*/*z* 306.1700 (calculated for C_17_H_24_NO_4_ is 306.1705).

***tert-butyl ((S)-4-methyl-1-((R)-2-methyloxiran-2-yl)-1-oxopentan-2-yl)carbamate*** (**8**). The product was synthesized according to the general procedure. ^1^H NMR (500 MHz, CDCl_3_) δ 4.81 (d, *J* = 8.9 Hz, 1H), 4.38–4.16 (m, 1H), 3.26 (d, *J* = 5.1 Hz, 1H), 2.86 (d, *J* = 5.0 Hz, 1H), 1.69 (m, 2H), 1.49 (m, 4H), 1.38 (s, 9H), 0.94 (d, *J* = 6.5 Hz, 3H), 0.90 (d, *J* = 6.7 Hz, 3H); ^13^C NMR (125 MHz, CDCl_3_) δ 209.8, 155.8, 79.9, 59.2, 52.5, 51.6, 40.6, 28.5, 25.3, 23.6, 21.5, 17.0. ESI-HRMS [M + H]^+^ *m*/*z* 272.1856 (calculated for C_14_H_26_NO_4_ is 272.1862).

***(S)-2-amino-3-(2,4-difluorophenyl)-1-((R)-2-methyloxiran-2-yl)propan-1-one*** (**9**). The product was synthesized according to the general procedure. ^1^H NMR (500 MHz, CDCl_3_) δ 7.23 (d, *J* = 13.3 Hz, 2H), 6.84 (dddd, J = 18.9, 10.8, 8.5, 2.5 Hz, 2H), 4.33 (t, *J* = 5.8 Hz, 1H), 3.26 (d, *J* = 5.5 Hz, 2H), 3.12 (d, *J* = 4.4 Hz, 1H), 2.96 (d, *J* = 4.3 Hz, 1H), 1.49 (s, 3H); ^13^C NMR (125 MHz, CDCl3) δ 204.1, 162.2, 161.4, 132.6, 115.5, 112.4, 104.6, 59.2, 53.6, 52.7, 29.7, 16.1. ESI-HRMS [M + H]^+^ *m*/*z* 242.0096 (calculated for C_12_H_12_F_2_NO_2_ is 242.0093).

***(S)-2-amino-3-(4-fluorophenyl)-1-((R)-2-methyloxiran-2-yl)propan-1-one*** (**10**). The product was synthesized according to the general procedure. ^1^H NMR (500 MHz, CDCl_3_) δ 7.36–7.28 (m, 3H), 7.19–7.14 (m, 2H), 4.33 (dd, *J* = 8.7, 4.2 Hz, 1H), 3.41 (dd, *J* = 14.6, 4.1 Hz, 1H), 3.14 (d, *J* = 4.4 Hz, 1H), 2.99 (d, *J* = 4.3 Hz, 1H), 2.94 (dd, *J* = 14.6, 8.6 Hz, 1H), 1.53 (s, 3H); ^13^C NMR (125 MHz, CDCl_3_) δ 204.2, 132.3, 129.5, 129.3, 128.5, 59.2, 55.7, 52.8, 36.0, 16.4. ESI-HRMS [M + H]^+^ *m*/*z* 206.1191 (calculated for C_12_H_16_NO_2_ is 206.1181).

***tert-butyl ((S)-3-(2,4-difluorophenyl)-1-((S)-2-methyloxiran-2-yl)-1-oxopropan-2-yl)carbamate*** (**11**). The product was synthesized according to the general procedure. ^1^H NMR (500 MHz, CDCl_3_) δ 7.10 (td, *J* = 8.5, 6.4 Hz, 1H), 6.84–6.74 (m, 2H), 4.94 (s, 1H), 4.67 (s, 1H), 3.02 (s, 0H), 2.99 (d, *J* = 4.9 Hz, 1H), 2.85 (d, *J* = 4.8 Hz, 1H), 2.78 (d, *J* = 10.7 Hz, 1H), 1.54 (s, 3H), 1.35 (s, 9H); ^13^C NMR (125 MHz, CDCl_3_) δ 207.9, 163.4, 162.5, 161.4, 160.5, 155.3, 132.5, 118.9, 111.6, 103.8, 80.2, 59.3, 52.9, 52.6, 30.5, 28.4, 16.6. ESI-HRMS [M + H]^+^ *m*/*z* 342.1522 (calculated for C_17_H_22_F_2_NO_4_ is 342.1517).

***tert-butyl ((S)-3-(4-fluorophenyl)-1-((S)-2-methyloxiran-2-yl)-1-oxopropan-2-yl)carbamate*** (**12**). The product was synthesized according to the general procedure. ^1^H NMR (500 MHz, CDCl_3_) δ 7.32–7.25 (m, 2H), 7.24–7.19 (m, 1H), 7.14–7.07 (m, 2H), 4.88 (s, 1H), 4.66 (s, 1H), 2.99 (dd, *J* = 13.5, 7.5 Hz, 1H), 2.87–2.78 (m, 1H), 2.66 (d, *J* = 4.9 Hz, 1H), 2.56 (d, *J* = 4.9 Hz, 1H), 1.48 (s, 3H), 1.38 (s, 9H); 13C NMR (125 MHz, CDCl_3_) δ 207.6, 159.7, 136.2, 129.5, 128.7, 127.1, 80.1, 59.0, 54.3, 51.9, 38.2, 28.3, 17.3. ESI-HRMS [M + H]^+^ *m*/*z* 306.1706 (calculated for C_17_H_24_NO_4_ is 306.1705).

**Synthesis of *N-((S)-3-(2,4-difluorophenyl)-1-((R)-2-methyloxiran-2-yl)-1-oxopropan-2-yl)dodecanamide*** (**3**). To a solution of compound **5** (600 mg, 1.84 mmol) in DCM (10 mL) was added lauric acid (368 mg, 1.84 mmol, 1 equiv.), DiPEA (593 mg, 4.5 mmol, 2.5 equiv.), and HBTU (697 mg, 1.84 mmol, 1 equiv.). The reaction mixture was stirred overnight at room temperature and evaporated under vacuum. The residue were resuspended in ethyl acetate, washed with 1 M HCl (3X), saturated NaHCO_3_ (2X), brine, and then dried using anhydrous magnesium sulfate. It was evaporated under vacuum and the crude product was purified by column chromatography (50–75% ethyl acetate/hexanes) to give product **3** (483 mg, 62% yield). ^1^H NMR (500 MHz, CDCl_3_) δ 7.09 (q, *J* = 7.9 Hz, 1H), 6.78 (m, 2H), 5.88 (d, *J* = 8.0 Hz, 1H), 4.85–4.73 (m, 1H), 3.28 (d, J = 4.9 Hz, 1H), 2.98 (tt, J = 14.4, 6.1 Hz, 2H), 2.89 (d, *J* = 4.9 Hz, 1H), 2.09 (t, *J* = 7.6 Hz, 2H), 1.65–1.55 (m, 2H), 1.49 (s, 3H), 1.22 (m, 16H), 0.85 (t, *J* = 6.8 Hz, 3H); δ ^13^C NMR (125 MHz, CDCl_3_) δ 207.5, 173.1, 163.2, 162.2, 161.2, 160.2, 132.3, 118.8, 111.5, 103.8, 59.2, 52.6, 51.6, 40.8, 36.4, 31.9, 29.6, 29.4, 29.3, 29.3, 29.1, 25.4, 22.7, 16.40, 14.1. ESI-HRMS [M + H]^+^ *m*/*z* 424.2661 (calculated for C_24_H_36_F_2_NO_3_ is 424.2663). 

**Synthesis of *N-((S)-1-(((S)-3-(2,4-difluorophenyl)-1-((R)-2-methyloxiran-2-yl)-1-oxopropan-2-yl)amino)-1-oxohexan-2-yl)dodecanamide* (4).** To a solution of compound **5** (338 mg, 1.0 mmol) in DCM (5 mL) was added Boc-Nle-OH (231 mg, 1.0 mmol, 1 equiv.), DiPEA (322 mg, 3.0 mmol, 2.5 equiv.), and HBTU (379 mg, 1.0 mmol, 1 equiv.). The reaction mixture was stirred overnight at room temperature and evaporated under vacuum. The residue were resuspended in ethyl acetate, washed with 1 M HCl (3X), saturated NaHCO_3_ (2X), brine, and then dried using anhydrous magnesium sulfate. It was evaporated under vacuum and the crude product was used in the next step without further purification. 

The Boc protecting group of the crude product was removed by dissolving the desired epoxyketone product in DCM:TFA (2:1) for 20 min, and the solvent evaporated to give the corresponding TFA amine salt. The product (1.0 mmol) was dissolved in DCM (5 mL) followed by addition of lauric acid (200 mg, 1.0 mmol, 1 equiv.), DiPEA (322 mg, 3.0 mmol, 2.5 equiv.), and HBTU (379 mg, 1.0 mmol, 1 equiv.). The reaction mixture was stirred overnight at room temperature and evaporated under vacuum. The residue were resuspended in ethyl acetate, washed with 1 M HCl (3X), saturated NaHCO_3_ (2X), brine, and then dried using anhydrous magnesium sulfate. It was evaporated under vacuum and the crude product was purified by column chromatography (50–75% ethyl acetate/hexanes) to give product **4** (257 mg, 48% yield). ^1^H NMR (500 MHz, CDCl_3_) δ 7.10 (m, 1H), 6.78 (m, 2H), 6.50 (d, *J* = 7.7 Hz, 1H), 5.85 (d, *J* = 7.9 Hz, 1H), 4.89–4.63 (m, 1H), 4.34 (m, 1H), 3.24 (d, *J* = 4.9 Hz, 1H), 3.01 (m, 2H), 2.90 (d, *J* = 4.8 Hz, 1H), 2.14 (t, *J* = 7.6 Hz, 2H), 1.71 (m, 2H), 1.64–1.56 (m, 2H), 1.49 (s, 3H), 1.24 (m, 20H), 0.85 (m, 6H). (126 MHz, CDCl_3_) δ 206.80, 173.18, 171.85, 163.30, 162.33, 161.42, 160.26, 132.50, 118.40, 111.43, 103.97, 59.36, 52.85, 52.61, 51.81, 36.70, 32.11, 31.99, 30.15, 29.70, 29.58, 29.42, 29.34, 27.54, 25.74, 22.77, 22.44, 16.48, 14.21, 13.95. ESI-HRMS [M + H]^+^ *m*/*z* 537.3506 (calculated for C_30_H_47_F_2_N_2_O_4_ is 537.3504). 

### 3.3. Anti-Lipase Activity

#### 3.3.1. Preparation of the Candidate Inhibitors for Biological Testing

All tested compounds were dissolved in DMSO to prepare a stock solution (of each compound) having a known concentration. A proper aliquot of each tasted compound was obtained by further dilution of the stock. Samples were prepared and analyzed for their phenyl acetate content by adopting an internal standard procedure. the sample analysis performed using GC-FID. For each sample, 50 µL of 2 mg/mL pancreatic lipase, phenyl acetate (1 mM), and 50 µL of each candidate inhibitor. For the reference sample and the sample containing the active enzyme 50 µL of water used in place of the inhibitor. Subsequently, the samples were left for overall time of 20 min vortex and centrifuged for approximately 3 min. For extraction of the phenyl acetate remain in the aqueous layer the nonpolar solvent chloroform containing the internal standard added, then vortex and centrifuged and the chloroform removed to another tube for the GC-FID analysis.

#### 3.3.2. GC-FID Method Validation

**Figure 5:**
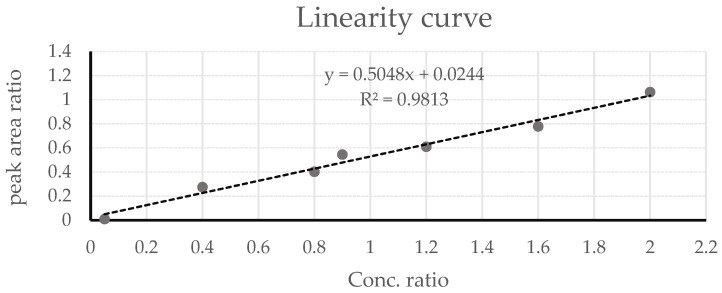
Linearity curve for phenyl acetate in the GC-FID-based antilipase assay.

The linearity, RSD, LOD, and LOQ were evaluated for the method of analysis. Under the instrumental operating conditions as described. phenyl acetate and its internal standard were perfectly separated with a relative standard division percentage (RSD%) of 5.6%. The LOD and LOQ both was measured using the signal-to-noise method. The ratio of the analyte signal to the peak-to-peak noise signal around it was measured of (3:1) which is generally accepted signal-to-noise ratio (S/N) for the LOD estimation. The lower limit of quantification measured at 25 µM of phenyl acetate and the lower limit of detection of phenyl acetate measured at 10 µM. Figure 5 shows the linearity curve for phenyl acetate in the GC-FID based anti-lipase assay. 

### 3.4. Cytotoxic Activity

All the cell line that were used in this study are (CCD-1064SK) the human normal skin fibroblast and HepG2 hepatoma cell line. The CCD-1064SK and HepG2 cell line was purchased from American Type Culture Collection (ATCC, Manassas, VA, USA). Cells were cultured in Iscove’s medium as recommended from ATCC. Media was supplement with 10% heated fetal bovine serum, 1% of 2 Mm L-glutamine, and (100 IU/mL) penicillin/(100 ug/mL) streptomycin. According to the cells growth profile, cells were seeded with a density of 10 × 10^4^ cell/well. Cell viability was determined by trypan blue exclusion using a hemocytometer. For the cytotoxicity assay, cells were washed with phosphate buffer saline (PBS). PBS was decanted and cells detached with 0.025% trypsin-EDTA (EuroClone). Media was added to a volume of 10 mL. The cell suspension was centrifuged at 1000 rpm for 10 min and the pellet was resuspended in 10 mL medium to make a single cell suspension. Viability of the cells was determined by trypan blue exclusion, and it exceeded 90% as counted in a hemocytometer. The cell suspension was diluted afterwards to give the optimal seeding density and 100 uL of the cell suspension was plated in a 96 well plate. Cells were cultured at 37 °C in a humidified atmosphere of 5% CO_2_. After 24 h the cells were treated with different concentrations of tested compounds, (each compound was initially dissolved in DMSO). Initially, 100 and 50 ug/mL of each extract were tested. The active compounds were those which gave less than 50% survival at exposure time 72 h. The final dilution for all prepared concentrations used for treating the cells contained not more than 0.01% of the initial solvent (DMSO), this concentration being used in the solvent control wells. At the end of the exposure time, cell growth was analyzed using the MTT assay. **MTT assay:** After 72 h of incubation, 15 µL of MTT stock solution (Promega) (5 mg/mL in sterile phosphate-buffered saline, pH 7.4) was added to each well. After the cells were incubated for 3 h in the presence of MTT, 100 µL of solubilizing stop solution was added to each well to solubilize the dark violet formazan crystals. The optical densities at 570 nm were then measured using a microplate reader. The results were expressed as a percentage of cell viability with respect to a control corresponding to untreated cells.

### 3.5. Molecular Docking 

Crystalline structure of human Pancreatic lipase was retrieved from RCSB protein data bank (PDB code: 1LPB) and prepared to suit docking analysis. Capping of both N and C-terminals was acquired pH for generating het states using Epik was set at 7.4 ± 0.2. The energy minimization was processed by OPLS3e force field in Mastero Schrödinger software suite. Grid file representing active site of protein was generated by choosing some common residues (e.g., Serine 152) in orlistat active site inside the crystalline structure of the protein as centroid through Glidgrid generation protocol. Ligands were drawn in ChemDraw and imported into Mastero as mol files, the ligands were prepared by ligprep command with ionization state at 7.4 ± 0.2. They were energetically minimized by OPLS3e force filed. Docking of the prepared ligands into the grid file of the protein was calculated with scaling factor of receptor van der Waals for nonpolar atoms at 0.8 to give better chance for receptor flexibility. Docking scores as well as ligand poses obtained from docking study were carefully evaluated to determine the in silico binding of the ligands with the protein.

## 4. Conclusions

It is reported here for the first time that the epoxyketone moiety represents a novel and potent anti-lipase warhead that can be used towards the development of anti-obesity drugs. Although it has been widely believed that epoxyketones are highly selective to the threonine in the active site of the proteasome, it is here demonstrated that epoxyketones can also inhibit lipase enzymes via an active site serine residue. Analogues **3** and **4** represent a highly lipophilic form of these lipase inhibitors, which likely enhances the cell permeability, absorption and other pharmacokinetic parameters. The truncated structure in analogues **5**–**8** shows that the lipase favors the epoxyketone irrespective of the amino acid nature, whether or not the side chain is aromatic. In addition, the configuration of the epoxyketone seems to play no clear role in the lipase inhibitory activity of these very small molecules. The free amine version of these amino acid-derived epoxyketones is as active as the Boc-protected analogues that we tested here. These data all show that the lipase enzyme is flexible and can interact with various types of epoxyketones, a characteristic that can be used to modulate the drug-like parameters of our novel anti-obesity drug leads. In vivo validation of these in vitro results and toxicity studies, along with further synthesis of structurally diverse epoxyketones and rigorous testing will be important for the anti-obesity drug development using these lead molecules as lipase inhibitors.

## Figures and Tables

**Table 1:** Evaluation of inhibitory activity of the synthesized analogues **3**–**13** against PL.

Analogue	IC_50_ (nM) against PL
orlistat (**1**)	100.8 ± 13.2
carmaphycin B (**2**)	25.0 ± 1.1
**3**	29.3 ± 2.4
**4**	9.3 ± 2.5
**5**	29.4 ± 4.8
**6**	46.4 ± 3.7
**7**	49.1 ± 11.4
**8**	120 ± 15.2
**9**	41.5 ± 7.1
**10**	73.1 ± 9.8
**11**	29.0 ± 4.2
**12**	49.2 ± 9.3
**13**	>10,000

**Table 2:** Cytotoxicity evaluation of the synthesized analogues **3**–**12** against fibroblast and HepG2 cell lines.

Compound Code	CCD-1064Sk Fibroblasts IC_50_ (nM)	HepG2 Cells IC_50_ (nM)
**3**	>15,000	2598
**4**	>15,000	2325
**5**	>15,000	>15,000
**6**	>15,000	>15,000
**7**	>15,000	>15,000
**8**	>15,000	>15,000
**9**	>15,000	>15,000
**10**	>15,000	>15,000
**11**	>15,000	>15,000
**12**	>15,000	>15,000
**13**	>15,000	>15,000

## Data Availability

Not applicable.

## Sample Availability

Samples of all synthesized compounds are available from the authors.

## References

[1] Blüher M. (2019). Obesity: Global Epidemiology and Pathogenesis. Nat. Rev. Endocrinol..

[2] Fabricatore A.N., Wadden T.A. (2006). Obesity. Annu. Rev. Clin. Psychol..

[3] Kyrou I., Randeva H.S., Tsigos C., Kaltsas G., Weickert M.O. (2018). Clinical Problems Caused by Obesity. *Endotext*. https://www.ncbi.nlm.nih.gov/books/NBK278973/.

[4] Kompaniyets L., Goodman A.B., Belay B., Freedman D.S., Sucosky M.S., Lange S.J., Gundlapalli A.V., Boehmer T.K., Blanck H.M. (2021). Body Mass Index and Risk for COVID-19–Related Hospitalization, Intensive Care Unit Admission, Invasive Mechanical Ventilation, and Death—United States, March–December 2020. MMWR Morb. Mortal. Wkly. Rep..

[5] Cai Z., Yang Y., Zhang J. (2021). Obesity Is Associated with Severe Disease and Mortality in Patients with Coronavirus Disease 2019 (COVID-19): A Meta-Analysis. BMC Public Health.

[6] Hall K.D., Kahan S. (2018). Maintenance of Lost Weight and Long-Term Management of Obesity. Med. Clin. N. Am..

[7] Chanoine J.P., Hampl S., Jensen C., Boldrin M., Hauptman J. (2005). Effect of Orlistat on Weight and Body Composition in Obese Adolescents: A Randomized Controlled Trial. JAMA.

[8] Bray G.A., Heisel W.E., Afshin A., Jensen M.D., Dietz W.H., Long M., Kushner R.F., Daniels S.R., Wadden T.A., Tsai A.G. (2018). The Science of Obesity Management: An Endocrine Society Scientific Statement. Endocr. Rev..

[9] Löffler M.C., Betz M.J., Blondin D.P., Augustin R., Sharma A.K., Tseng Y.H., Scheele C., Zimdahl H., Mark M., Hennige A.M. (2021). Challenges in Tackling Energy Expenditure as Obesity Therapy: From Preclinical Models to Clinical Application. Mol. Metab..

[10] Wang H., Eckel R.H. (2009). Lipoprotein Lipase: From Gene to Obesity. Am. J. Physiol. Endocrinol. Metab..

[11] Borrelli G.M., Trono D. (2015). Recombinant Lipases and Phospholipases and Their Use as Biocatalysts for Industrial Applications. Int. J. Mol. Sci..

[12] Liu T.T., Liu X.T., Chen Q.X., Shi Y. (2020). Lipase Inhibitors for Obesity: A Review. Biomed. Pharmacother..

[13] Guerra J.V.S., Dias M.M.G., Brilhante A.J.V.C., Terra M.F., García-Arévalo M., Figueira A.C.M. (2021). Multifactorial Basis and Therapeutic Strategies in Metabolism-Related Diseases. Nutrients.

[14] Eichmann T.O., Lass A. (2015). DAG Tales: The Multiple Faces of Diacylglycerol—Stereochemistry, Metabolism, and Signaling. Cell. Mol. Life Sci..

[15] Heck A.M., Yanovski J.A., Calis K.A. (2000). Orlistat, a New Lipase Inhibitor for the Management of Obesity. Pharmacotherapy.

[16] Lee M.J., Bhattarai D., Yoo J., Miller Z., Park J.E., Lee S., Lee W., Driscoll J.J., Kim K.B. (2019). Development of Novel Epoxyketone-Based Proteasome Inhibitors as a Strategy to Overcome Cancer Resistance to Carfilzomib and Bortezomib. J. Med. Chem..

[17] Huang X., Dixit V.M. (2016). Drugging the Undruggables: Exploring the Ubiquitin System for Drug Development. Cell Res..

[18] Almaliti J., Miller B., Pietraszkiewicz H., Glukhov E., Naman C.B., Kline T., Hanson J., Li X., Zhou S., Valeriote F.A. (2019). Exploration of the Carmaphycins as Payloads in Antibody Drug Conjugate Anticancer Agents. Eur. J. Med. Chem..

[19] Kubiczkova L., Pour L., Sedlarikova L., Hajek R., Sevcikova S. (2014). Proteasome Inhibitors-Molecular Basis and Current Perspectives in Multiple Myeloma. J. Cell. Mol. Med..

[20] Itoh K., Matsukawa T., Murata K., Nishitani R., Yamagami M., Tomohiro N., Kajiyama I., Fumuro M., Iijima M., Shigeoka S. (2019). Pancreatic Lipase Inhibitory Activity of Citrus Unshiu Leaf Extract. Nat. Prod. Commun..

[21] Pereira A.R., Kale A.J., Fenley A.T., Byrum T., Debonsi H.M., Gilson M.K., Valeriote F.A., Moore B.S., Gerwick W.H. (2012). The Carmaphycins: New Proteasome Inhibitors Exhibiting an α,β-Epoxyketone Warhead from a Marine Cyanobacterium. ChemBioChem.

[22] Sankar V., Maida Engels S. (2018). Synthesis, Biological Evaluation, Molecular Docking and in Silico ADME Studies of Phenacyl Esters of N-Phthaloyl Amino Acids as Pancreatic Lipase Inhibitors. Future J. Pharm. Sci..

[23] Nguyen P.T.V., Huynh H.A., van Truong D., Tran T.D., Vo C.V.T. (2020). Exploring Aurone Derivatives as Potential Human Pancreatic Lipase Inhibitors through Molecular Docking and Molecular Dynamics Simulations. Molecules.

[24] Kisselev A.F., Van Der Linden W.A., Overkleeft H.S. (2012). Proteasome Inhibitors: An Expanding Army Attacking a Unique Target. Chem. Biol..

[25] LaMonte G.M., Almaliti J., Bibo-Verdugo B., Keller L., Zou B.Y., Yang J., Antonova-Koch Y., Orjuela-Sanchez P., Boyle C.A., Vigil E. (2017). Development of a Potent Inhibitor of the Plasmodium Proteasome with Reduced Mammalian Toxicity. J. Med. Chem..

[26] Almaliti J., Fajtová P., O’Donoghue A.J., AlHindy M., Gerwick W.H. (2021). Improved Scalable Synthesis of Clinical Candidate KZR-616, a Selective Immunoproteasome Inhibitor. ChemistrySelect.

